# Supplementary material for: Identification of photoperiod-induced specific miRNAs in the adrenal glands of Sunite sheep (Ovis aries)
Source: Front Vet Sci. 2022 Jul 22;9:888207. doi: 10.3389/fvets.2022.888207 (PMC9354845; doi:10.3389/fvets.2022.888207)
Supplement: Supplementary file 1 [file Data_Sheet_1.ZIP › Supplementary file/miRDeep2 file.zip!/miRDeep2 file/Know_Structure/LP42A3/oar-mir-376e.pdf]

miRBase precursor : oar-mir-376e
Total read count : 1330
oar-miR-376e-5p read count : 278
oar-miR-376e-3p read count : 774
oar-miR-376b-5p read count : 278
remaining reads : 0

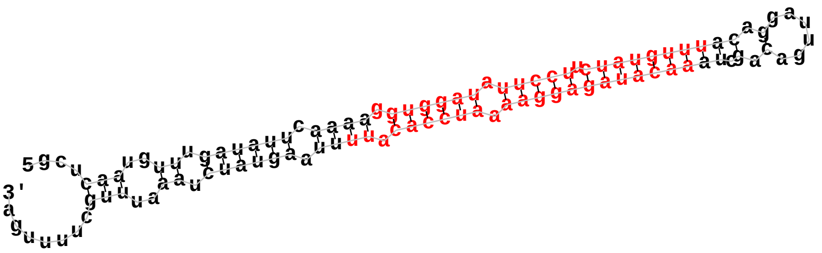

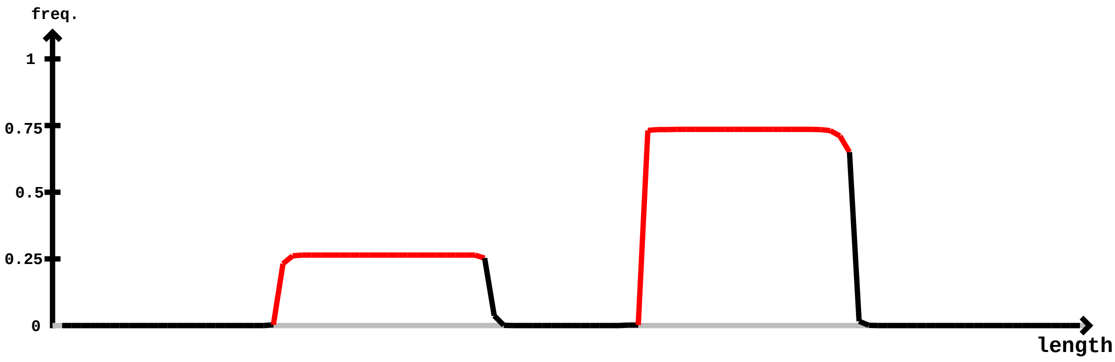


```
                                                            oar-miR-376e-3p
                      oar-miR-376b-5p
                      oar-miR-376e-5p
5'- gcucaauguuugauauucaaaagguggauauuccuucuauguuuacaggauugacagcuaaacauagaggaaaauccacauuuuaaguaucuaaauuugcuuuuga   -3'    exp
    ((.....(((((((((.((((.((((((.(((((.((((((((((.(......).).))))))))))))).)))))).)))).)))))).)))).))......    reads      mm         sample
    .....................agguggauauuccuucuauguuu..............................................................    3          0          seq
    ......................gguggauauuccuucuauguu...............................................................    11         0          seq
    ......................gguggauauuccuucuauguuu..............................................................    197        0          seq
    ......................gguggauauuccuucuauguuua.............................................................    32         0          seq
    ......................gguggauauuccuucuauguuuac............................................................    1          0          seq
    .......................guggauauuccuucuauguu...............................................................    1          0          seq
    .......................guggauauuccuucuauguuu..............................................................    25         0          seq
    .......................guggauauuccuucuauguuua.............................................................    5          0          seq
    ........................uggauauuccuucuauguuu..............................................................    2          0          seq
    ........................uggauauuccuucuauguuua.............................................................    1          0          seq
    ..........................................................uaaacauagaggaaaauccaca..........................    2          0          seq
    ............................................................aacauagaggaaaaucca............................    1          0          seq
    ............................................................aacauagaggaaaauccac...........................    4          0          seq
    ............................................................aacauagaggaaaauccaca..........................    19         0          seq
    ............................................................aacauagaggaaaauccacau.........................    64         0          seq
    ............................................................aacauagaggaaaauccacauu........................    664        0          seq
    ............................................................aacauagaggaaaauccacauuu.......................    15         0          seq
    ............................................................aacauagaggaaaauccacauuuu......................    1          0          seq
    .............................................................acauagaggaaaauccacauu........................    2          0          seq
    .............................................................acauagaggaaaauccacauuu.......................    1          0          seq
    ...............................................................auagaggaaaauccacauu........................    1          0          seq
```
